# The Role of New Technologies in Myeloproliferative Neoplasms

**DOI:** 10.3389/fonc.2019.00321

**Published:** 2019-04-26

**Authors:** Giuseppe A. Palumbo, Stefania Stella, Maria Stella Pennisi, Cristina Pirosa, Elisa Fermo, Sonia Fabris, Daniele Cattaneo, Alessandra Iurlo

**Affiliations:** ^1^Department of Scienze Mediche, Chirurgiche e Tecnologie Avanzate “G.F. Ingrassia,” University of Catania, Catania, Italy; ^2^Center of Experimental Oncology and Hematology, A.O.U. Policlinico-Vittorio Emanuele, Catania, Italy; ^3^Department of Clinical and Experimental Medicine, University of Catania, Catania, Italy; ^4^Postgraduate School of Hematology, University of Catania, Catania, Italy; ^5^Hematology Division, Foundation IRCCS Ca' Granda Ospedale Maggiore Policlinico, Milan, Italy; ^6^Hematology Division, Myeloproliferative Syndromes Unit, Foundation IRCCS Ca' Granda Ospedale Maggiore Policlinico, Milan, Italy

**Keywords:** *BCR-ABL1*-negative myeloproliferative neoplasms (MPNs), myelofibrosis (MF), *JAK2* mutations, calreticulin (*CALR*), *MPL* (W515K/L), *ASXL1* mutation, High molecular risk (HMR) mutations, next generation sequencing (NGS)

## Abstract

The hallmark of *BCR-ABL1*-negative myeloproliferative neoplasms (MPNs) is the presence of a driver mutation in *JAK2, CALR*, or *MPL* gene. These genetic alterations represent a key feature, useful for diagnostic, prognostic and therapeutical approaches. Molecular biology tests are now widely available with different specificity and sensitivity. Recently, the allele burden quantification of driver mutations has become a useful tool, both for prognostication and efficacy evaluation of therapies. Moreover, other sub-clonal mutations have been reported in MPN patients, which are associated with poorer prognosis. *ASXL1* mutation appears to be the worst amongst them. Both driver and sub-clonal mutations are now taken into consideration in new prognostic scoring systems and may be better investigated using next generation sequence (NGS) technology. In this review we summarize the value of NGS and its contribution in providing a comprehensive picture of mutational landscape to guide treatment decisions. Finally, discussing the role that NGS has in defining the potential risk of disease development, we forecast NGS as the standard molecular biology technique for evaluating these patients.

## Introduction

Myeloproliferative neoplasms (MPNs) are clonal disorders of the hematopoietic stem cell, mainly characterized by proliferative bone marrow with varying degrees of reticulin/collagen fibrosis, extramedullary hematopoiesis, abnormal peripheral blood count, and constitutional symptoms that are secondary to abnormally expressed inflammatory cytokines (1). Among the so-called “*BCR-ABL1*-negative MPNs” polycythemia vera (PV), essential thrombocythemia (ET) and primary myelofibrosis (PMF) are included. However, unlike chronic myeloid leukemia (which is always characterized by the *BCR–ABL1* fusion gene), they have not yet been associated with any specific genetic abnormalities.

The discovery in 2005 of the *JAK2*V617F point mutation (2–5) and the subsequent identification of other specific abnormalities, such as *JAK2* exon 12 (6, 7), *MPL* exon 10 (8–10) and *CALR* exon 9 (11, 12), gave an improvement in understanding their genetic basis. All of them are now included in the molecular diagnostic and prognostic algorithms for MPNs, leading to several revisions of the diagnostic criteria for these diseases. In addition, they are used as markers of disease burden and as a measure of assessing response to various therapeutic interventions that can target the mutant clone.

The MPN driver mutations are often mutually exclusive and, after the detection of the common *JAK2*V617F mutation, generally no further testing is performed. Nevertheless, in recent years, several reports have suggested that driver mutations indeed do rarely coexist (13–16), but additional studies are needed to clarify the clinical implications of double-mutated cases.

Importantly, additional sub-clonal driver and non-driver mutations in genes such as *ASXL1, SRSF2, EZH2, IDH1*, and *IDH2*, among others, have been identified as being associated with disease progression (17, 18). A wide choice of techniques is currently available for the detection of MPN mutations, and a continuous evolution of molecular diagnostic applications and platforms is now ongoing.

### JAK2

**Figure 1:**
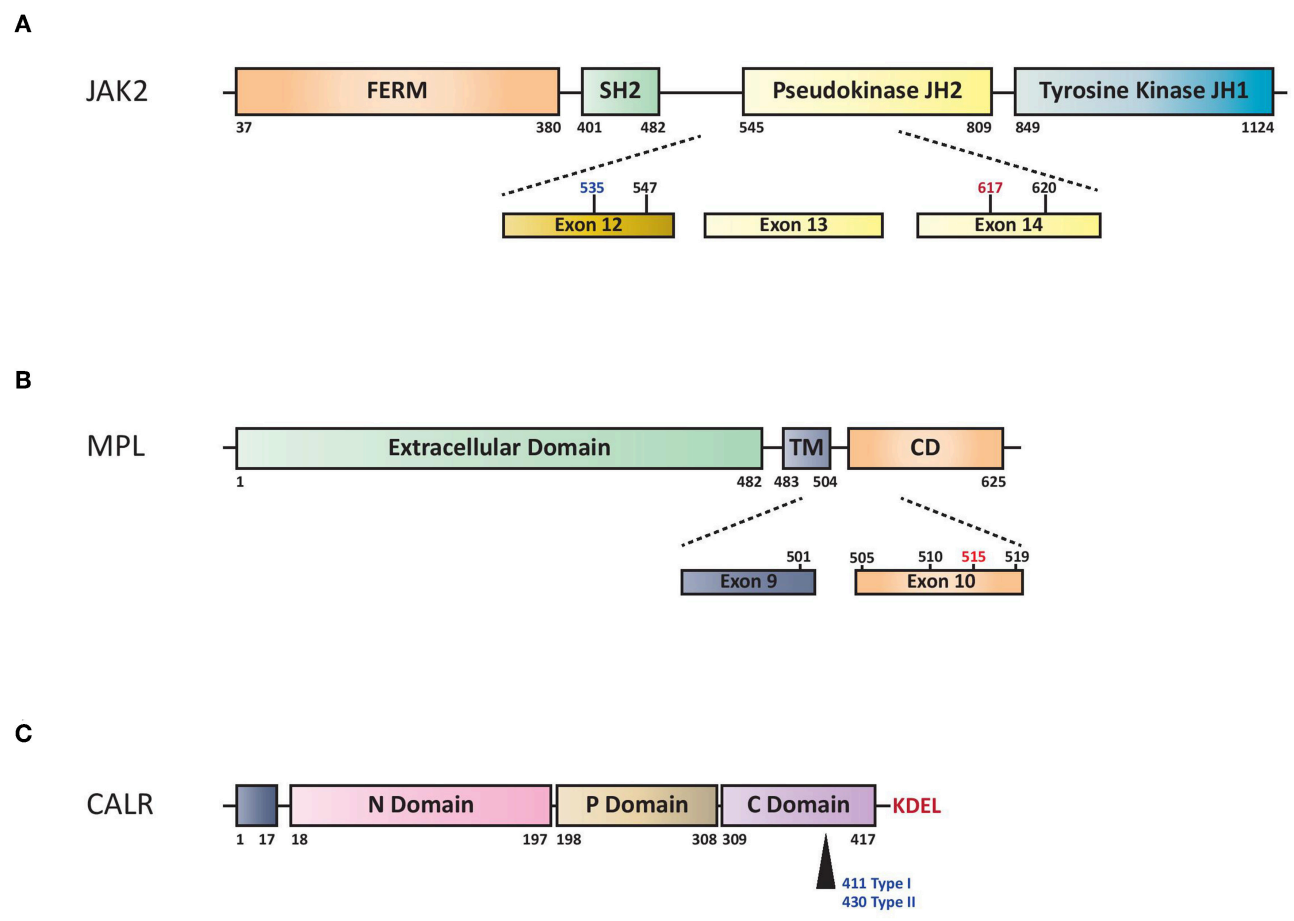
**(A)** Schematic representation of structural domains of the *JAK2*. FERM indicates 4.1 Ezrin, Radixin, Moesin domain; JH1 and JH2 signal (JAK Homology 1 and 2) domains refer to tyrosine kinase and pseudo-kinase domains, respectively. SH2 indicates Src Homology 2 domain. Numbers represent amino acid positions within the *JAK2* protein: red indicates V617F mutation, in black other SNP mutations and in blue the region for insertions/deletions. **(B)** Structural domains and mutations of the MPL. Extracellular domain indicates NH2 amino terminal region, TM transmembrane domain and CD cytoplasmic domain. Dashed lines highlights exons reported mutations of MPL. W515L or W515K are indicated in red while the others hot spots variant mutations in black. Numbers represent amino acid positions. **(C)** Structural domains of CALR protein. CALR includes NH2 domain (N-Domain) and Proline-rich domain (P-Domain) with chaperone lectin-like function. COOH domain (C Domain) has aminoacids responsible for Ca^++^-buffering. Arrow indicates Type I (deletion) or Type II (insertion) alterations that determine loss of KDEL aminoacids and generation of a new tail with low calcium buffering.

*JAK2* is a non-receptor tyrosine kinase, which, upon ligand binding to specific cytokine receptors, is phosphorylated and activated, leading to regulation of gene expression involved in cell proliferation and survival. The *JAK2*V617F mutation is a G to T somatic mutation at nucleotide 1849 in exon 14, resulting in the substitution of valine to phenylalanine at codon 617, which triggers constitutive activation of downstream signaling and uncontrolled cell growth (Figure 1A).

V617F mutation in exon 14 of *JAK2* gene is present in the majority of patients with PV (more than 95%) and in 60% of those with ET or PMF (2–5). Rare insertions and deletions in exon 12 have been described in 2 to 3% of patients with PV (6, 7) (Figure 1A). The most widely used method for *JAK2*V617F detection is based on allele-specific PCR (2). Quantitative PCR methods (qPCR) are preferred over qualitative ones because of greater reproducibility and sensitivity and because of the need of quantifying the mutated clone in MPN patients.

Actually, V617F allelic burden at diagnosis provides important prognostic information, being found to be associated with phenotypic presentation and severity of MPNs (19–21), the risk of thrombotic events (20, 22) and progression to secondary myelofibrosis (MF) (23, 24). In particular, in PMF patients *JAK2*V617F mutation is associated with clinical characteristics which include older age, higher hemoglobin level, leukocytosis, and lower platelet count (20; (25) and a low *JAK2*V617F allele burden may represent a favorable prognostic factor (26). With regards to PV, a higher *JAK2*V617F mutant allele burden has been associated with more frequent thrombotic complications (20), pruritus and fibrotic transformation (27). Moreover, V617F allelic burden measured during the follow-up is currently used in the course of treatment with alpha-interferon and *JAK1/2* inhibitors (28–30), as well as for minimal residual disease (MRD) evaluation after allogeneic stem cells transplantation (31). In fact, although the ideal therapy should be able to eradicate the malignant MPN clone, this aim has not been reached with the current available treatments in contrast to the striking efficacy of tyrosine kinase inhibitor monotherapy in chronic myeloid leukemia (32).

Several quantitative methods have been developed in the years, most of them based on real-time allele-specific PCR, with sensitivity ranging from 0.05 to 1%, and specificity of 100% (33–36). Digital PCR (dPCR) has also been proposed with the aim to achieve an absolute quantification of the target gene without the need for a standard curve, with comparable or higher performances compared to qPCR (37–39). Recently, Next Generation Sequencing (NGS) has been shown to allow the detection of the V617F mutation with comparable performances, but weaker sensitivity to qPCR, with the advantage of detection of new potentially pathogenic *JAK2* variants (40).

Regarding *JAK2* exon 12 variants detection, different approaches can be adopted; Sanger Sequencing (SS) is the most frequently used because of the rarity and heterogeneity of these mutations (41, 42). Nested High-Resolution Melting (HRM) curve analysis has been proposed as highly sensitive screening method eventually followed by SS for the precise characterization of the mutation (36, 43).

### MPL

The myeloproliferative leukemia virus oncogene (*MPL*) is located on chromosome 1p34 and encodes for the thrombopoietin (TPO) receptor, thus assuming a crucial role in the regulation of megakaryocyte growth and survival. In 2006, a somatic activating mutation in exon 10 of this gene, *MPL*W515L, was described in *JAK2*V617F-negative ET/PMF (44, 45). This mutation is characterized by a G to T transition at nucleotide 1544, resulting in a tryptophan to leucine substitution at codon 515 of the transmembrane region of *MPL*, inducing constitutive activation of the TPO receptor in a cytokine-independent fashion (Figure 1B).

Activating mutations in *MPL* are reported in ~5–10% of all PMF patients and 1–4% of those with ET (44–46). All of them cluster in exon 10 and in the majority of cases affect a tryptophan in position 515 (W515L, W515K, W515A, and W515R).

The methods applied for the detection of *MPL* mutations can be grouped according to different strategies: targeted identification of specific mutations or sequencing of the entire exon 10. Targeted analysis include allele-specific PCR, allele-specific qPCR and Amplification Refractory Mutation System (ARMS)-PCR; among them qPCR displays the highest sensitivity (0.1–0.5%) (8, 9, 47–49). A multiplexed allele-specific PCR assay for the four most frequent *MPL* exon 10 mutations (W515L, W515K, W515A, and S505N) has been developed, with 100% specificity and 2.5% sensitivity (50). Analysis of the entire exon 10 allows the detection of all the known and potentially new mutations and can be achieved by SS, pyrosequencing or HRM curve. The sensitivity of these approaches is lower, reaching 2–5% for the latter method (10, 51).

### Calreticulin

Calreticulin (*CALR*) mutations were reported for the first time at the end of 2013 (11, 12). These mutations represent the second most common genetic abnormality in MPNs after *JAK2*V617F, even though they are absent in PV patients. On the other hand, *CALR* mutations partially addressed the molecular gap in *JAK2*/*MPL*-unmutated ET and PMF patients, accounting for 20–25% of the overall somatic mutations. The remaining cases (i.e., negative for *JAK2, CALR*, and *MPL*) are termed “triple-negative,” representing the 5–10% of all *BCR-ABL1*-negative MPN patients.

*CALR* is a multi-functional Ca2+ binding protein with chaperone activity mainly localized in the endoplasmic reticulum. The localization and retention of *CALR* are defined by the N-terminal signal sequence and the C-terminal ER-retention sequence KDEL (Figure 1C). It is involved in numerous intracellular (cytoplasm and nucleus), cell surface, and extracellular functions such as protein quality control, calcium metabolism, immune response, phagocytosis, cell adhesion and others (52). *CALR* mutations were phenotypic drivers in the pathogenesis of MPNs (53). Recently, studies concerning the role of *CALR* mutated proteins demonstrated that they are able to bind to the *MPL* receptor inducing JAK-STAT activation and the positive aminoacid charge of the mutant C terminus is required to mediate this interaction (54–57).

More than 50 different *CALR* mutations have been described so far, with type 1 (L367fs^*^46) resulted from 52-bp deletion and type 2 (K385fs^*^47) from 5-bp TTGTC insertion accounting for ~80% of all the cases. More type 1 (53%) than type 2 (32%) abnormalities are found in MPN patients (11), whereas the remaining cases are classified as either type 1-like, type 2-like, or “other type,” based on their structural similarities to the classical mutations. Their distribution is 57% type 1(-like) and 39% type 2(-like) in ET and 83% type 1(-like) and 15% type 2(-like) in PMF (58). All these abnormalities frequently consist of insertions or deletions involving exon 9 of the gene, generating a frameshift to a unique alternative reading frame; it results in a novel C-terminus peptide sequence enriched for positively charged residues. Furthermore, the mutated protein lacks the KDEL signal, leading to a partial dislocation of *CALR* from the endoplasmic reticulum (11).

In PMF patients the favorable prognostic impact is limited to *CALR* type 1/type 1-like mutations, whereas type 2/type 2-like are associated with a worse prognosis, similar to that of *JAK2*-positive patients (59). On the contrary, in ET *CALR* type 1 and type 1-like mutations are associated with an higher risk of MF transformation (58).

As previously published for *JAK2* mutations, association between *CALR* mutation allele burden and disease phenotype has been reported (60, 61). In particular, an association between *CALR* allele burden, leukocyte and platelet counts, hemoglobin and lactate dehydrogenase levels was described. Furthermore, the median *CALR* allele burden remained steady over time; interestingly, differently from *JAK2*-positive cases, acute myeloid leukemias (AML) evolving from *CALR*-mutant MPNs commonly maintained their mutational profile (61). Additional studies are needed to better clarify the *CALR* mutant allele burden clinical implications.

Given their high frequency in MPNs, screening for *CALR* mutations is recommended in all *JAK2*V617F-negative patients with suspected ET or PMF (36, 62, 63). Several methods have been proposed to detect *CALR* mutations, including SS, fragment length analysis, real-time qPCR, HRM, ddPCR, pyrosequencing and NGS (64–69). Due to the heterogeneity of *CALR* insertions and deletions detected, direct SS is considered to be the primary method of testing. Indeed, SS of exon 9, providing specific sequence change information, allowed to identify the exact type of *CALR* mutation and detailed procedures, described in previous publications, were the most used conditions in diagnostic routine screening laboratories.

### Role of Next-Generation Sequencing in MPNs

Nowadays, NGS has played an important role in understanding the genetic alterations of different human cancers. There are several number of available NGS platforms using different sequencing technologies, which perform sequencing of millions of small fragments of DNA in parallel (70). Nevertheless, different sequencing chemistry and methods for signal detection, the obtained results are comparable. Bioinformatics analyses are used to piece together these fragments by mapping the individual reads to the human reference genome.

This method provides several advantages compared to different sequencing methods. First of all, NGS is a high-throughput method as it detects concomitant mutations in the same run. Then, the analysis requires low input of DNA/RNA sample as compared to traditional sequencing methods (e.g., SS or Pyrosequencing). Moreover, NGS discriminates genomic aberrations, which are screened simultaneously, such as single/multiple nucleotide variants (SNVs), small and large insertions and deletions (ins/dels) and copy-number variations (CNV) with high sensitivity and accuracy, so reducing data analysis and clinical reporting time (Table 1) (70).

**Table 1:** Comparison of Real Time PCR, Digital PCR, SS, and NGS technologies in clinical molecular diagnostics.

	**Benefits**	**Critical points**	**Sensitivity**
Real time PCR	Detection of known mutations Validated Methods	High input of DNA/RNA No simultaneous screening of multiple genes in multiple samples	1%
Sanger sequencing	Detection of known and unknown mutations Validated methods	High input of DNA/RNA	10–20%
Digital PCR	Low input of DNA/RNA Detection of known mutations Cost-effective for rapid genotyping e monitoring	No simultaneous screening of multiple genes in multiple samples	0.1–1%
NGS	Low input of DNA/RNA Massively parallel sequencing Decreased sequencing cost/gene Detection of known and unknown mutations Simultaneous screening of multiple genes in multiple samples	Validation studies require High-complexity workflow and analyzing results Genome data analysis is time-consuming	1%

*PCR, Polymerase Chain Reaction; SS, Sanger Sequencing; NGS, Next Generation Sequencing*.

In the last few years, NGS have been applied to many hematological disorders such as for establishing T-cell clonality, recurrent cytogenetic translocations and prognosis of Philadelphia chromosome-positive acute lymphoblastic leukemia (ALL) (70). In addition, NGS approaches can be used in diagnostic samples to understand the possible evolution of MRD in clonal *IGH* and *TCR* rearrangements in lymphoproliferative disorders (71). A comparison study between the most common technologies used to lymphoproliferative disorders, underlined that NGS method was able to identify mutant or clonal DNA in few tumor circulating cells. For this reason, NGS may be used to improve MRD in post-therapy monitoring (72).

In MPNs, it was demonstrated that *JAK2* V617F allele burden identified patients with different clinical course: in PV and in ET, variant allele frequency of *JAK2* V617F, was associated with a higher ratio of fibrotic transformation. In patients in PMF high variant allele frequency was correlated to recurrent thrombosis event, while low variant allele frequency was associated to short leukemia-free survival and overall survival, probably due to acquisition of additional driver mutations. In fact, new NGS panels are used as predict methods to describe alterations of several genes involved in transcriptional regulation and cell signaling pathways (Table 2). These information can be used to predict the risk leukemic transformation, to guide target treatment and to speculate a personalized clinical trial (32, 73, 84). Using the high sensitivity and specificity of NGS technology, several groups analyzed and deeply studied the genetic and molecular profile of MPN subjects, with the aim of assessing not only non-driver mutations, but also infrequent variants of driver mutations (Table 2). In MPN patients negative for *JAK2, CALR*, and *MPL* (triple-negative, TN), mutations in *LNK, TET2, DNMT3A, IDH1/2, CBL*, and *ASXL1* genes but also atypical mutation in *MPL* (S204P) have been identified (83, 85). In this contest, Chang *et al*. identified 30 missense mutations in 12 of 16 triple-negative MPN patients. In particular, in this study NGS platform was able to identify low allelic burden and atypical mutations of *JAK2* (*JAK2*V626F and *JAK2*F556V) and *MPL* (*MPL*S204P and *MPL*Y591N) (86).

**Table 2:** Mutated genes in *BCR-ABL1*-negative myeloproliferative neoplasms (MPNs).

**Gene**	**Pathway relevance**	**Type of mutation**	**Frequency of mutation (%, PV, ET, MF)**	**Prognostic significance**	**References**
ASXL1	Epigenetic regulation	Missense	3–12% in PV 4–11% in ET 22–38% in MF	Adverse in PV and MF	(17, 18, 25, 30, 32, 45, 73–82)
DNMT3A	Epigenetic regulation	Missense	6% in ET 5–10% in MF	None	(18, 32, 73, 74, 76, 77, 82)
EZH2	Epigenetic regulation	Missense	2–12% in PV 3% in ET 12% in MF	Adverse in TE and MF	(18, 25, 32, 73–77, 79, 80, 82)
IDH1	Epigenetic regulation	Missense	10% in PV 1% in ET 1–4% in MF	Adverse in MF	(18, 25, 32, 74–77, 79, 80, 82)
IDH2	Epigenetic regulation	Missense	4% in PV 1–3% in MF	Adverse in PV and MF	(18, 25, 32, 74–77, 79, 80, 82)
TET2	Epigenetic regulation	Insertion/ DeletionNonsense or Missense	10–25% in PV 16% in ET 17% in MF	Adverse in TE	(18, 32, 73, 74, 76, 77, 82)
SF3B1	mRNA processing	Missense	3% in PV 5% in ET 10% in MF	Adverse in TE	(18, 32, 74, 77, 82)
SRSF2	mRNA processing	Missense	9% in MF <2% ET	Adverse in PV and MF	(18, 25, 32, 74–77, 80–82)
U2AF1	mRNA processing	Missense	1–2% in TE 10–17% in MF	Adverse in TE and MF	(18, 32, 74, 77, 82)
ZRSR2	mRNA processing	Missense	5% in PV 3% in ET 10% in MF	Not known	(18, 32, 74, 77, 82)
CEBPA	Transcriptional regulation	Mutations	6% in PV 4% in ET 9% in MF	Adverse in MF	(18, 32, 82)
RUNX1	Transcriptional regulation	Nonsense Missense Insertion/ Deletion	<5% (PV, ET, MF)	Adverse in MF	(18, 32, 74, 76, 77, 82)
TP53	Transcriptional regulation	Missense or Mutation	<5% (PV, ET, MF)	Adverse in TE	(18, 32, 74, 76, 77, 82)
CBL	Cell signaling pathways	Missense	4% in MF	Adverse in MF	(18, 32, 73, 74, 77, 82)
KIT	Cell signaling pathways	Mutations	3% in PV 2% in ET 1% in MF	Adverse in MF	(18, 32, 82)
NF1	Cell signaling pathways	Deletion	Rare in MF	Not known	(18, 32, 74, 77, 82)
NRAS/KRAS	Cell signaling pathways	Missense	1% in ET 1–4% in MF	Not known	(18, 32, 74, 77, 82)
SH2B3/LNK	Cell signaling pathways	Deletion or missense	9% in PV 3% in ET 11–18% in MF	Adverse in TE and MF	(18, 32, 73, 74, 77, 82, 83)

*For each gene the function, type of molecular alteration, frequency and prognostic significance, if known, are reported*.

Furthermore, Cabagnols et al. noticed that an atypical mutation of *MPL* (*MPL*S204P) was associated with other alterations in *ASXL1* and *SRSF2* genes. The subjects with more than one alteration were classified as myelodysplastic syndrome (MDS) with thrombocytosis rather than ET. The *MPL*S204P is a weak gain-of-function mutation that induces constitutive STAT activation and more prolonged ligand-induced STAT phosphorylation than wild-type *MPL*, while *MPL*Y591N is associated with *MPL*W515A and in a mouse model induced a more aggressive MPN behavior than that associated with a single *MPL*Y591N mutant (87).

Additionally, applying NGS methodology on a group of 197 MPN patients, Lundberg *et al*. observed several alterations concerning genes involved in DNA methylation (*TET2, DNMT3A, IDH1*) and chromatin structure (*EZH2, ASXL1)* regulation. Moreover, they analyzed the impact of somatic mutations on clinical outcomes and found that the concomitant somatic mutations in *TP53* or *TET2* were correlated to both reduction of overall survival (OS) and increased risk of leukemic evolution (88). Also Agarwal et al., using a customized 26-gene NGS panel in a series of 171 MPN patients, highlighted that alterations in *ASXL1, EZH2*, and *IDH1/2* were associated with an increasing risk of disease progression and a shorter OS in both ET and PMF patients. In this work the authors found that among *JAK2*-mutated patients, 88% of subjects presented a single *JAK2* mutation while the remaining 12% showed additional mutations in *TET2, ASXL1*, and *SF3B1* genes.

Finally, very recently Grinfeld *et al*. (89) sequencing coding exons from 69 myeloid cancer genes in a large and representative series of 2035 MPN patients, identified different genetic subgroups with distinct clinical phenotypes, including blood counts, risk of leukemic transformation, and event-free survival. Integration of 63 clinical and genomic variables allowed the authors to develop a prognostic model (https://cancer.sanger.ac.uk/mpn-multistage/) capable of generating personally tailored predictions of clinical outcomes in MPN patients, even within individual categories of existing prognostic schemas. In particular, they identified a first subgroup with *TP53* disruption or aneuploidy characterized by a poor outcomes and high risk of transformation to AML; a second subgroup with chromatin or spliceosome mutations that showed an increased risk for transformation to MF and shorter event-free survival. Patients who are not included in these two subgroups were defined according to their dominant phenotypic driver mutation and were the following: patients with *CALR, MPL*, heterozygous *JAK2*, and homozygous *JAK2* or *NFE2* mutations. The remaining two subgroups included instead patients with no detectable driver mutations and those with additional driver mutations not identified in the other six subgroups. Thus, the model provides considerable discriminatory power that accurately generalizes to other real-world cohorts.

Focusing on *CALR*-mutated cases, 56/62 (90%) of subjects showed single mutation in *CALR* gene while 10% had additional mutations in *TET2* and *ASXL1* (*CALR* type 1 mutations were more represented in this cohort of patients than type 2 mutations). On the contrary, no additional mutations were noticed in *MPL*-mutated patients (90). In addition, Song *et al*. analyzed 135 MPN patients by NGS and found that *JAK2, ASXL1*, and *TET2* were frequently mutated in PMF, PV, and ET; interestingly, the comparison between mutational and cytogenetic profiles identified a possibility role in triaging and guiding different treatments (91).

Different studies have demonstrated the utility of NGS technologies to analyze the genetic profiles in AML patients. This technique is applied not only to study primary but also secondary AML, a heterogeneous group of disorders arising from MDS or MDS/MPN. In fact, secondary AMLs are characterized by several cytogenetic abnormalities and genetic alterations. In particular, Hussaini et al. (92), examining 187 subjects with a diagnosis of AML, highlighted the frequency of related-gene mutations, where *ASXL1* was the highest mutated gene, followed by *TET2, RUNX1, DNMT3A, TP53, IDH2, NRAS, FLT3*, and *NPM1*. Moreover, NGS analysis identified co-mutated genes (*ASXL1* with *RUNX1* or *TET2* or *NRAS*) and was able to discriminate somatic mutations associated with MDS/AML.

The prognostic impact of certain co-occurring mutations has been associated with MPNs disease progression as well as with the development of secondary AML (74, 93). Accordingly, the value of NGS at present lays also in the risk stratification of leukemic evolutions, in particular in critical and difficult cases, thus guiding treatment decision. In this respect, in PMF patients *ASXL1* seems to be the worst sub-clonal mutation (25, 45). Indeed, the presence of this mutation or any other among *SRSF2* and *IDH1/2* gives a High Molecular Risk (HMR) to the patient and is included in the Mutation-Enhanced International Prognostic Score System (MIPSS70) and MIPSS70-plus, where the number of HMR mutations is also weighted together with driver mutation status and clinical data. The presence of two or more HMR mutations has been associated with highly adverse prognosis and rapid leukemic transformation (75, 76). The association of HMR and worse prognosis has also been confirmed in a retrospective series of elderly MF patients (77). Similarly, in the Genetically Inspired Prognostic Scoring System (GIPSS), only the molecular features are taken into consideration and *IDH1/2* are substituted by *U2AF1* mutations (78). In the same way, in ET and PV patients *IDH2, U2AF1, EZH2, TP53, SH2B3*, and *SF3B1* mutations were all reported to give an adverse prognostic value (93).

Finally, somatic mutations with frequency ranging from 10 to 20% have been described in elderly subjects without any clinical evidence of myeloid diseases. In 2016, Bartels et al. used a customized NGS panel with 23 genes mutated in both MDS and MPNs to profile 192 formalin-fixed and paraffin-embedded (FFPE) patient samples. In this study, the authors found overall 269 pathogenic mutations in 125 of 185 analyzed patients and several of these exhibited more one-gene variants. Although they used FFPE bone marrow samples, the study demonstrated that NGS improved diagnostic accuracy and contributed to understanding the development, progression and therapy of myeloid diseases (94). NGS technology provide additional information to define the potential risk of development a myeloid malignancy, understand the clinical course, select the appropriate target therapy and predict potential drug-resistance mechanisms.

## Conclusions

The discovery of *JAK2*V617F mutation in *BCR-ABL1*-negative MPNs by four different international cooperative groups in 2005 (2–5) led to significant insights on the pathogenesis of these disorders. In fact, this mutation results in a gain-of-function with activation of cytokine and growth factor receptors, and thus of the downstream JAK-STAT pathway (79, 95–98). The *JAK2* point mutation in exon 12, present in a small percentage of patients with PV, is able to induce the MPN phenotype through the same pathogenic mechanism (6, 7).

In 2006 the *MPL*W515L/K was reported in ET and PMF patients (44, 45) and demonstrated to be able to aberrantly activate JAK-STAT pathway through a gain-of function similar to that of *JAK2*, thus leading to megakaryocytic proliferation (8–10).

More recently, in 2013 two different groups demonstrated a spectrum of mutations in *CALR* gene that cause frameshifts of one base pair in the last coding exon with a generation of a protein with new C terminus and a tail of 36 aminoacids (11, 12). Although it was soon clear that JAK-STAT signaling pathway was consistently activated in *CALR* mutated cells (53, 99), only recently it has been demonstrated that the mutant *CALR* protein is able to bind to the *MPL* receptor and activate it independently of the TPO presence itself (54–57). Consistently with the fact that all these mutations cause JAK-STAT pathway activation, ruxolitinib, the first *JAK1/2* inhibitor, is able to exert clinical results in MF patients independently of the type of mutation (80).

As *BCR-ABL1*-negative MPNs are initiated by a driver mutation in one of *JAK2, CALR* or *MPL* genes in more than 95% of cases, there is a key relevance in diagnosis, prognosis and therapy (12, 61, 81). Due to the fact that these mutations are mutually exclusive, a sequential search for diagnosing purposes starting from *JAK2* is suggested (36, 62–64). Furthermore, each of these mutations has a different prognostic impact. In ET, *JAK2* is taken into consideration in the IPSET prognostic scores (100, 101). In *JAK2*-positive PMF, allele burden is relevant, with a worse prognosis for patients with < 50%, while type 1/type 1-like *CALR* mutations has a favorable impact (22, 26). In the new MYSEC-PM prognostic score developed for secondary MF, *CALR* mutations absence is associated with a worse outcome (102).

Since NGS studies have been performed in *BCR-ABL1*-negative MPNs, especially in PMF, other mutations than the driver ones have been reported with few particularly relevant for prognosis and prediction of response. Sub-clonal mutations in genes of the spliceosome machinery, regulators of chromatin structure and histone modification and epigenetic regulators of DNA methylation are now taken into consideration in the new prognostic scores together with driver mutation status and clinical data in MIPSS70 and MIPSS70-plus (75) or in the GIPSS, where only the molecular features are evaluated (45).

NGS analysis during the disease natural history is of key importance to underline the eventual clonal evolution, thus uncovering an aggressive course and possibly suggesting a change in the therapeutic strategy (103, 104). Thus, in a near future, due to the progressive lowering of costs and availability in more reference laboratories, NGS will become the standard to evaluate *BCR-ABL1*-negative MPN patients.

## Author Contributions

All authors listed have made a substantial, direct and intellectual contribution to the work, and approved it for publication.

### Conflict of Interest Statement

The authors declare that the research was conducted in the absence of any commercial or financial relationships that could be construed as a potential conflict of interest.

## References

[1] Arber DA Orazi A Hasserjian R Thiele J Borowitz MJ Le Beau MM. The 2016 revision to the World Health Organization classification of myeloid neoplasms and acute leukemia. Blood. (2016) 127:2391–405. 10.1182/blood-2016-03-643544 27069254

[2] Baxter EJ Scott LM Campbell PJ East C Fourouclas N Swanton S. Acquired mutation of the tyrosine kinase *JAK2* in human myeloproliferative disorders. Lancet. (2005) 365:1054–61. 10.1016/S0140-6736(05)71142-9 15781101

[3] Kralovics R Passamonti F Buser AS Teo SS Tiedt R Passweg JR. A gain-of-function mutation of *JAK2* in myeloproliferative disorders. N Engl J Med. (2005) 352:1779–90. 10.1056/NEJMoa051113 15858187

[4] Levine RL Wadleigh M Cools J Ebert BL Wernig G Huntly BJ. Activating mutation in the tyrosine kinase *JAK2* in polycythemia vera, essential thrombocythemia, and myeloid metaplasia with myelofibrosis. Cancer Cell. (2005) 7:387–97. 10.1016/j.ccr.2005.03.023 15837627

[5] James C Ugo V Le Couedic JP Staerk J Delhommeau F Lacout C. A unique clonal *JAK2* mutation leading to constitutive signalling causes polycythaemia vera. Nature. (2005) 434:1144–8. 10.1038/nature03546 15793561

[6] Scott LM Tong W Levine RL Scott MA Beer PA Stratton MR. *JAK2* exon 12 mutations in polycythemia vera and idiopathic erythrocytosis. N Engl J Med. (2007) 356:459–68. 10.1056/NEJMoa065202 17267906 PMC2873834

[7] Pietra D Li S Brisci A Passamonti F Rumi E Theocharides A. Somatic mutations of *JAK2* exon 12 in patients with *JAK2* (V617F)-negative myeloproliferative disorders. Blood. (2008) 111:1686–9. 10.1182/blood-2007-07-101576 17984312

[8] Beer PA Campbell PJ Scott LM Bench AJ Erber WN Bareford D. MPL mutations in myeloproliferative disorders: analysis of the PT-1 cohort. Blood. (2008) 112:141–9. 10.1182/blood-2008-01-131664 18451306

[9] Pancrazzi A Guglielmelli P Ponziani V Bergamaschi G Bosi A Barosi G. A sensitive detection method for MPLW515L or MPLW515K mutation in chronic myeloproliferative disorders with locked nucleic acid-modified probes and real-time polymerase chain reaction. J Mol Diagnost JMD. (2008) 10:435–41. 10.2353/jmoldx.2008.080015 18669880 PMC2518738

[10] Schnittger S Bacher U Haferlach C Beelen D Bojko P Burkle D Characterization of 35 new cases with four different MPLW515 mutations and essential thrombocytosis or primary myelofibrosis. Haematologica. (2009) 94:141–4. 10.3324/haematol.13224 19029146 PMC2625421

[11] Klampfl T Gisslinger H Harutyunyan AS Nivarthi H Rumi E Milosevic JD. Somatic mutations of calreticulin in myeloproliferative neoplasms. N Engl J Med. (2013) 369:2379–90. 10.1056/NEJMoa1311347 24325356

[12] Nangalia J Massie CE Baxter EJ Nice FL Gundem G Wedge DC. Somatic CALR mutations in myeloproliferative neoplasms with nonmutated *JAK2*. N Engl J Med. (2013) 369:2391–405. 10.1056/NEJMoa1312542 24325359 PMC3966280

[13] Mansier O Migeon M Etienne G Bidet A Lippert E. *JAK2*V617F and CALR double mutations are more frequently encountered in patients with low *JAK2*V617F allelic burdens. Leukemia Lymphoma. (2016) 57:1949–51. 10.3109/10428194.2015.1116122 26727159

[14] Usseglio F Beaufils N Calleja A Raynaud S Gabert J. Detection of CALR and MPL mutations in low allelic burden *JAK2* V617F essential thrombocythemia. J Mol Diagnost JMD. (2017) 19:92–8. 10.1016/j.jmoldx.2016.08.006 27855276

[15] Nussenzveig RH Pham HT Perkins SL Prchal JT Agarwal AM Salama ME. Increased frequency of co-existing *JAK2* exon-12 or MPL exon-10 mutations in patients with low *JAK2*(V617F) allelic burden. Leukemia Lymphoma. (2016) 57:1429–35. 10.3109/10428194.2015.1091932 26419289

[16] Haunstrup LM Ebbesen LH Hansen M Severinsen MT Aggerholm A. Skewed ratio between type 1 and type 2 calreticulin mutations in essential thrombocytosis patients with concomitant Janus kinase 2 V617F mutation. Exp Hematol. (2018) 68:62–5. 10.1016/j.exphem.2018.09.007 30292681

[17] Tefferi A Guglielmelli P Lasho TL Rotunno G Finke C Mannarelli C. CALR and ASXL1 mutations-based molecular prognostication in primary myelofibrosis: an international study of 570 patients. Leukemia. (2014) 28:1494–500. 10.1038/leu.2014.57 24496303

[18] Tefferi A Lasho TL Finke CM Elala Y Hanson CA Ketterling RP. Targeted deep sequencing in primary myelofibrosis. Blood Adv. (2016) 1:105–11. 10.1182/bloodadvances.2016000208 29296803 PMC5737166

[19] Larsen TS Pallisgaard N Moller MB Hasselbalch HC. The *JAK2* V617F allele burden in essential thrombocythemia, polycythemia vera and primary myelofibrosis–impact on disease phenotype. Eur J Haematol. (2007) 79:508–15. 10.1111/j.1600-0609.2007.00960.x 17961178

[20] Vannucchi AM Antonioli E Guglielmelli P Pardanani A Tefferi A. Clinical correlates of *JAK2*V617F presence or allele burden in myeloproliferative neoplasms: a critical reappraisal. Leukemia. (2008) 22:1299–307. 10.1038/leu.2008.113 18496562

[21] Perricone M Palandri F Ottaviani E Angelini M Bagli L Bellesia E. Assessment of the interlaboratory variability and robustness of *JAK2*V617F mutation assays: a study involving a consortium of 19 Italian laboratories. Oncotarget. (2017) 8:32608–17. 10.18632/oncotarget.15940 28427233 PMC5464813

[22] Guglielmelli P Barosi G Specchia G Rambaldi A Lo Coco F Antonioli E. Identification of patients with poorer survival in primary myelofibrosis based on the burden of *JAK2*V617F mutated allele. Blood. (2009) 114:1477–83. 10.1182/blood-2009-04-216044 19549988

[23] Koren-Michowitz M Landman J Cohen Y Rahimi-Levene N Salomon O Michael M. *JAK2*V617F allele burden is associated with transformation to myelofibrosis. Leukemia Lymphoma. (2012) 53:2210–3. 10.3109/10428194.2012.682308 22524513

[24] Latagliata R Polverelli N Tieghi A Palumbo GA Breccia M Sabattini E Comparison of *JAK2*(V617F) -positive essential thrombocythaemia and early primary myelofibrosis: the impact of mutation burden and histology. Hematol Oncol. (2018) 36:269–75. 10.1002/hon.2430 28509339

[25] Vannucchi AM Lasho TL Guglielmelli P Biamonte F Pardanani A Pereira A. Mutations and prognosis in primary myelofibrosis. Leukemia. (2013) 27:1861–9. 10.1038/leu.2013.119 23619563

[26] Rozovski U Verstovsek S Manshouri T Dembitz V Bozinovic K Newberry K. An accurate, simple prognostic model consisting of age, *JAK2*, CALR, and MPL mutation status for patients with primary myelofibrosis. Haematologica. (2017) 102:79–84. 10.3324/haematol.2016.149765 27686378 PMC5210235

[27] Passamonti F Rumi E Pietra D Elena C Boveri E Arcaini L. A prospective study of 338 patients with polycythemia vera: the impact of *JAK2* (V617F) allele burden and leukocytosis on fibrotic or leukemic disease transformation and vascular complications. Leukemia. (2010) 24:1574–9. 10.1038/leu.2010.148 20631743

[28] Kiladjian JJ Cassinat B Chevret S Turlure P Cambier N Roussel M. Pegylated interferon-alfa-2a induces complete hematologic and molecular responses with low toxicity in polycythemia vera. Blood. (2008) 112:3065–72. 10.1182/blood-2008-03-143537 18650451

[29] Quintas-Cardama A Abdel-Wahab O Manshouri T Kilpivaara O Cortes J Roupie AL. Molecular analysis of patients with polycythemia vera or essential thrombocythemia receiving pegylated interferon alpha-2a. Blood. (2013) 122:893–901. 10.1182/blood-2012-07-442012 23782935 PMC3739035

[30] Vannucchi AM Verstovsek S Guglielmelli P Griesshammer M Burn TC Naim A. Ruxolitinib reduces *JAK2* p.V617F allele burden in patients with polycythemia vera enrolled in the RESPONSE study. Ann Hematol. (2017) 96:1113–20. 10.1007/s00277-017-2994-x 28456851 PMC5486779

[31] Kroger N Badbaran A Holler E Hahn J Kobbe G Bornhauser M. Monitoring of the *JAK2*-V617F mutation by highly sensitive quantitative real-time PCR after allogeneic stem cell transplantation in patients with myelofibrosis. Blood. (2007) 109:1316–21. 10.1182/blood-2006-08-039909 17018857

[32] Greenfield G McPherson S Mills K McMullin MF. The ruxolitinib effect: understanding how molecular pathogenesis and epigenetic dysregulation impact therapeutic efficacy in myeloproliferative neoplasms. J Transl Med. (2018) 16:360. 10.1186/s12967-018-1729-7 30558676 PMC6296062

[33] Lippert E Girodon F Hammond E Jelinek J Reading NS Fehse B. Concordance of assays designed for the quantification of *JAK2*V617F: a multicenter study. Haematologica. (2009) 94:38–45. 10.3324/haematol.13486 19001280 PMC2625411

[34] Merker JD Jones CD Oh ST Schrijver I Gotlib J Zehnder JL. Design and evaluation of a real-time PCR assay for quantification of *JAK2* V617F and wild-type *JAK2* transcript levels in the clinical laboratory. J Mol Diagnostics JMD. (2010) 12:58–64. 10.2353/jmoldx.2010.090068 19959796 PMC2797719

[35] Jovanovic JV Ivey A Vannucchi AM Lippert E Oppliger Leibundgut E Cassinat B. Establishing optimal quantitative-polymerase chain reaction assays for routine diagnosis and tracking of minimal residual disease in *JAK2*-V617F-associated myeloproliferative neoplasms: a joint European LeukemiaNet/MPN&MPNr-EuroNet (COST action BM0902) study. Leukemia. (2013) 27:2032–9. 10.1038/leu.2013.219 23860450 PMC3806250

[36] Guglielmelli P Pietra D Pane F Pancrazzi A Cazzola M Vannucchi AM. Recommendations for molecular testing in classical Ph1-neg myeloproliferative disorders-A consensus project of the Italian Society of Hematology. Leukemia Res. (2017) 58:63–72. 10.1016/j.leukres.2017.04.006 28460339

[37] Waterhouse M Follo M Pfeifer D von Bubnoff N Duyster J Bertz H. Sensitive and accurate quantification of *JAK2* V617F mutation in chronic myeloproliferative neoplasms by droplet digital PCR. Ann Hematol. (2016) 95:739–44. 10.1007/s00277-016-2623-0 26931113

[38] Fontanelli G Barate C Ciabatti E Guerrini F Grassi S Del Re M. Real-Time PCR and Droplet Digital PCR: two techniques for detection of the *JAK2*(V617F) mutation in Philadelphia-negative chronic myeloproliferative neoplasms. Int J Lab Hematol. (2015) 37:766–73. 10.1111/ijlh.12404 26189968

[39] Link-Lenczowska D Pallisgaard N Cordua S Zawada M Czekalska S Krochmalczyk D. A comparison of qPCR and ddPCR used for quantification of the *JAK2* V617F allele burden in Ph negative MPNs. Ann Hematol. (2018) 97:2299–308. 10.1007/s00277-018-3451-1 30056580 PMC6208664

[40] Maslah N Verger E Schlageter MH Miclea JM Kiladjian JJ Giraudier S. Next-generation sequencing for *JAK2* mutation testing: advantages and pitfalls. Ann Hematol. (2018). 10.1007/s00277-018-3499-y 30259120

[41] Passamonti F Elena C Schnittger S Skoda RC Green AR Girodon F. Molecular and clinical features of the myeloproliferative neoplasm associated with *JAK2* exon 12 mutations. Blood. (2011) 117:2813–6. 10.1182/blood-2010-11-316810 21224469

[42] Furtado LV Weigelin HC Elenitoba-Johnson KS Betz BL. A multiplexed fragment analysis-based assay for detection of *JAK2* exon 12 mutations. J Mol Diagnost JMD. (2013) 15:592–9. 10.1016/j.jmoldx.2013.04.006 23810504

[43] Carillo S Henry L Lippert E Girodon F Guiraud I Richard C. Nested high-resolution melting curve analysis a highly sensitive, reliable, and simple method for detection of *JAK2* exon 12 mutations–clinical relevance in the monitoring of polycythemia. J Mol Diagnost JMD. (2011) 13:263–70. 10.1016/j.jmoldx.2010.12.002 21497288 PMC3077738

[44] Pikman Y Lee BH Mercher T McDowell E Ebert BL Gozo M. MPLW515L is a novel somatic activating mutation in myelofibrosis with myeloid metaplasia. PLoS Med. (2006) 3:e270. 10.1371/journal.pmed.0030270 16834459 PMC1502153

[45] Tefferi A. Primary myelofibrosis: 2019 update on diagnosis, risk-stratification and management. Am J Hematol. (2018) 93:1551–60. 10.1002/ajh.25230 30039550

[46] Pardanani AD Levine RL Lasho T Pikman Y Mesa RA Wadleigh M. MPL515 mutations in myeloproliferative and other myeloid disorders: a study of 1182 patients. Blood. (2006) 108:3472–6. 10.1182/blood-2006-04-018879 16868251

[47] Pietra D Brisci A Rumi E Boggi S Elena C Pietrelli A. Deep sequencing reveals double mutations in cis of MPL exon 10 in myeloproliferative neoplasms. Haematologica. (2011) 96:607–11. 10.3324/haematol.2010.034793 21228032 PMC3069239

[48] Ghaderi M Stromberg O Porwit A. Rapid real-time PCR assay for detection of MPL W515L mutation in patients with chronic myeloproliferative disorders. Int J Lab Hematol. (2010) 32(1 Pt 2):122–6. 10.1111/j.1751-553X.2008.01118.x 19016916

[49] Zhuge J Zhang W Zhang W Xu M Hoffman R. Sensitive detection of MPLW515L/K mutations by amplification refractory mutation system (ARMS)-PCR. Clin Chim Acta Int J Clin Chem. (2010) 411(1-2):122–3. 10.1016/j.cca.2009.10.012 19852952

[50] Furtado LV Weigelin HC Elenitoba-Johnson KS Betz BL. Detection of MPL mutations by a novel allele-specific PCR-based strategy. J Mol Diagnost JMD. (2013) 15:810–8. 10.1016/j.jmoldx.2013.07.006 23994117

[51] Boyd EM Bench AJ Goday-Fernandez A Anand S Vaghela KJ Beer P. Clinical utility of routine MPL exon 10 analysis in the diagnosis of essential thrombocythaemia and primary myelofibrosis. Br J Haematol. (2010) 149:250–7. 10.1111/j.1365-2141.2010.08083.x 20151976

[52] Gold LI Eggleton P Sweetwyne MT Van Duyn LB Greives MR Naylor SM. Calreticulin: non-endoplasmic reticulum functions in physiology and disease. FASEB J Offic Publ Federation Am Soc Exp Biol. (2010) 24:665–83. 10.1096/fj.09-145482 19940256 PMC2830142

[53] Marty C Pecquet C Nivarthi H El-Khoury M Chachoua I Tulliez M. Calreticulin mutants in mice induce an MPL-dependent thrombocytosis with frequent progression to myelofibrosis. Blood. (2016) 127:1317–24. 10.1182/blood-2015-11-679571 26608331

[54] Chachoua I Pecquet C El-Khoury M Nivarthi H Albu RI Marty C. Thrombopoietin receptor activation by myeloproliferative neoplasm associated calreticulin mutants. Blood. (2016) 127:1325–35. 10.1182/blood-2015-11-681932 26668133

[55] Elf S Abdelfattah NS Chen E Perales-Paton J Rosen EA Ko A. Mutant calreticulin requires both its mutant C-terminus and the thrombopoietin receptor for oncogenic transformation. Cancer Disc. (2016) 6:368–81. 10.1158/2159-8290.CD-15-1434 26951227 PMC4851866

[56] Araki M Yang Y Masubuchi N Hironaka Y Takei H Morishita S. Activation of the thrombopoietin receptor by mutant calreticulin in CALR-mutant myeloproliferative neoplasms. Blood. (2016) 127:1307–16. 10.1182/blood-2015-09-671172 26817954

[57] Elf S Abdelfattah NS Baral AJ Beeson D Rivera JF Ko A. Defining the requirements for the pathogenic interaction between mutant calreticulin and MPL in MPN. Blood. (2018) 131:782–6. 10.1182/blood-2017-08-800896 29288169 PMC5814933

[58] Pietra D Rumi E Ferretti VV Di Buduo CA Milanesi C Cavalloni C. Differential clinical effects of different mutation subtypes in CALR-mutant myeloproliferative neoplasms. Leukemia. (2016) 30:431–8. 10.1038/leu.2015.277 26449662 PMC4740452

[59] Tefferi A Lasho TL Tischer A Wassie EA Finke CM Belachew AA. The prognostic advantage of calreticulin mutations in myelofibrosis might be confined to type 1 or type 1-like CALR variants. Blood. (2014) 124:2465–6. 10.1182/blood-2014-07-588426 25301336 PMC4192754

[60] Oh Y Song IC Kim J Kwon GC Koo SH Kim SY. Pyrosequencing-based quantitative measurement of CALR mutation allele burdens and their clinical implications in patients with myeloproliferative neoplasms. Clin Chim Acta Int J Clin Chem. (2018) 483:183–91. 10.1016/j.cca.2018.05.001 29727699

[61] Szuber N Tefferi A. Driver mutations in primary myelofibrosis and their implications. Curr Opin Hematol. (2018) 25:129–35. 10.1097/MOH.0000000000000406 29256926

[62] Mesa R Jamieson C Bhatia R Deininger MW Gerds AT Gojo I. Myeloproliferative Neoplasms, Version 2.2017, NCCN Clinical Practice Guidelines in Oncology. J Natl Comprehensive Cancer Netw JNCCN. (2016) 14:1572–611 27956542 10.6004/jnccn.2016.0169 PMC11807340

[63] Busque L Porwit A Day R Olney HJ Leber B Ethier V. Laboratory investigation of Myeloproliferative Neoplasms (MPNs): recommendations of the canadian Mpn group. Am J Clin Pathol. (2016) 146:408–22. 10.1093/ajcp/aqw131 27686169

[64] Tefferi A Pardanani A. Genetics: CALR mutations and a new diagnostic algorithm for MPN. Nat Rev Clin Oncol. (2014) 11:125–6. 10.1038/nrclinonc.2014.16 24514146

[65] Maier CL Fisher KE Jones HH Hill CE Mann KP Zhang L. Development and validation of CALR mutation testing for clinical diagnosis. Am J Clin Pathol. (2015) 144:738–45. 10.1309/AJCPXPA83MVCTSOQ 26486738

[66] Mehrotra M Luthra R Singh RR Barkoh BA Galbincea J Mehta P. Clinical validation of a multipurpose assay for detection and genotyping of CALR mutations in myeloproliferative neoplasms. Am J Clin Pathol. (2015) 144:746–55. 10.1309/AJCP5LA2LDDNQNNC 26486739

[67] Murugesan G Guenther-Johnson J Mularo F Cook JR Daly TM. Validation of a molecular diagnostic assay for CALR exon 9 indels in myeloproliferative neoplasms: identification of coexisting *JAK2* and CALR mutations and a novel 9 bp deletion in CALR. Int J Lab Hematol. (2016) 38:284–97. 10.1111/ijlh.12484 27018326

[68] Jones AV Ward D Lyon M Leung W Callaway A Chase A. Evaluation of methods to detect CALR mutations in myeloproliferative neoplasms. Leukemia Res. (2015) 39:82–7. 10.1016/j.leukres.2014.11.019 25499808

[69] Mansier O Migeon M Saint-Lezer A James C Verger E Robin M. Quantification of the mutant CALR allelic burden by digital PCR: application to minimal residual disease evaluation after bone marrow transplantation. J Mol Diagnost JMD. (2016) 18:68–74. 10.1016/j.jmoldx.2015.07.007 26596525

[70] Serrati S De Summa S Pilato B Petriella D Lacalamita R Tommasi S. Next-generation sequencing: advances and applications in cancer diagnosis. OncoTargets Ther. (2016) 9:7355–65. 10.2147/OTT.S99807 27980425 PMC5144906

[71] Ladetto M Bruggemann M Monitillo L Ferrero S Pepin F Drandi D. Next-generation sequencing and real-time quantitative PCR for minimal residual disease detection in B-cell disorders. Leukemia. (2014) 28:1299–307. 10.1038/leu.2013.375 24342950

[72] Gazzola A Mannu C Rossi M Laginestra MA Sapienza MR Fuligni F. The evolution of clonality testing in the diagnosis and monitoring of hematological malignancies. Therapeutic Adv Hematol. (2014) 5:35–47. 10.1177/2040620713519729 24688753 PMC3949299

[73] Saeidi K. Myeloproliferative neoplasms: current molecular biology and genetics. Crit Rev Oncol Hematol. (2016) 98:375–89. 10.1016/j.critrevonc.2015.11.004 26697989

[74] Vainchenker W Kralovics R. Genetic basis and molecular pathophysiology of classical myeloproliferative neoplasms. Blood. (2017) 129:667–79. 10.1182/blood-2016-10-695940 28028029

[75] Guglielmelli P Lasho TL Rotunno G Mudireddy M Mannarelli C Nicolosi M. MIPSS70: Mutation-enhanced international prognostic score system for transplantation-age patients with primary myelofibrosis. J Clin Oncol Offic J Am Soc Clin Oncol. (2018) 36:310–8. 10.1200/JCO.2017.76.4886 29226763

[76] Patel KP Newberry KJ Luthra R Jabbour E Pierce S Cortes J. Correlation of mutation profile and response in patients with myelofibrosis treated with ruxolitinib. Blood. (2015) 126:790–7. 10.1182/blood-2015-03-633404 26124496 PMC4528066

[77] Palandri F Catani L Bonifacio M Benevolo G Heidel F Palumbo GA. Ruxolitinib in elderly patients with myelofibrosis: impact of age and genotype. A multicentre study on 291 elderly patients. Br J Haematol. (2018) 183:35–46. 10.1111/bjh.15497 30010187

[78] Tefferi A Lasho TL Finke C Gangat N Hanson CA Ketterling RP. Prognostic significance of ASXL1 mutation types and allele burden in myelofibrosis. Leukemia. (2018) 32:837–9. 10.1038/leu.2017.318 29089644

[79] Mascarenhas J Roper N Chaurasia P Hoffman R. Epigenetic abnormalities in myeloproliferative neoplasms: a target for novel therapeutic strategies. Clin Epigenet. (2011) 2:197–212. 10.1007/s13148-011-0050-6 22704337 PMC3365400

[80] Guglielmelli P Biamonte F Rotunno G Artusi V Artuso L Bernardis I. Impact of mutational status on outcomes in myelofibrosis patients treated with ruxolitinib in the COMFORT-II study. Blood. (2014) 123:2157–60. 10.1182/blood-2013-11-536557 24458439

[81] Passamonti F Mora B Maffioli M. New molecular genetics in the diagnosis and treatment of myeloproliferative neoplasms. Curr Opin Hematol. (2016) 23:137–43. 10.1097/MOH.0000000000000218 26825696

[82] Tefferi A Lasho TL Guglielmelli P Finke CM Rotunno G Elala Y. Targeted deep sequencing in polycythemia vera and essential thrombocythemia. Blood Adv. (2016) 1:21–30. 10.1182/bloodadvances.2016000216 29296692 PMC5744051

[83] Pardanani A Lasho T Finke C Oh ST Gotlib J Tefferi A LNK mutation studies in blast-phase myeloproliferative neoplasms, and in chronic-phase disease with TET2, IDH, *JAK2* or MPL mutations. Leukemia. (2010) 24:1713–8. 10.1038/leu.2010.163 20724988

[84] Sallman DA Padron E. Integrating mutation variant allele frequency into clinical practice in myeloid malignancies. Hematol Oncol Stem Cell Ther. (2016) 9:89–95. 10.1016/j.hemonc.2016.04.003 27187622

[85] Tefferi A Pardanani A. Myeloproliferative Neoplasms: a contemporary review. JAMA Oncol. (2015) 1:97–105. 10.1001/jamaoncol.2015.89 26182311

[86] Chang YC Lin HC Chiang YH Chen CG Huang L Wang WT. Targeted next-generation sequencing identified novel mutations in triple-negative myeloproliferative neoplasms. Med Oncol. (2017) 34:83. 10.1007/s12032-017-0944-z 28389907

[87] Cabagnols X Favale F Pasquier F Messaoudi K Defour JP Ianotto JC. Presence of atypical thrombopoietin receptor (MPL) mutations in triple-negative essential thrombocythemia patients. Blood. (2016) 127:333–42. 10.1182/blood-2015-07-661983 26450985

[88] Gardner JA Peterson JD Turner SA Soares BL Lancor CR Dos Santos LL. Detection of CALR mutation in clonal and nonclonal hematologic diseases using fragment analysis and next-generation sequencing. Am J Clin Pathol. (2016) 146:448–55. 10.1093/ajcp/aqw129 27686171

[89] Grinfeld J Nangalia J Baxter EJ Wedge DC Angelopoulos N Cantrill R. Classification and personalized prognosis in myeloproliferative neoplasms. N Engl J Med. (2018) 379:1416–30. 10.1056/NEJMoa1716614 30304655 PMC7030948

[90] Agarwal R Blombery P McBean M Jones K Fellowes A Doig K. Clinicopathological differences exist between CALR- and *JAK2*-mutated myeloproliferative neoplasms despite a similar molecular landscape: data from targeted next-generation sequencing in the diagnostic laboratory. Ann Hematol. (2017) 96:725–32. 10.1007/s00277-017-2937-6 28161773

[91] Song J Hussaini M Zhang H Shao H Qin D Zhang X. Comparison of the mutational profiles of primary myelofibrosis, polycythemia vera, and essential thrombocytosis. Am J Clin Pathol. (2017) 147:444–52. 10.1093/ajcp/aqw222 28419183 PMC5402718

[92] Hussaini MO Mirza AS Komrokji R Lancet J Padron E Song J. Genetic landscape of acute myeloid leukemia interrogated by next-generation sequencing: a large cancer center experience. Cancer Genom Proteom. (2018) 15:121–6. 10.21873/cgp.20070 29496691 PMC5892606

[93] Bacher U Shumilov E Flach J Porret N Joncourt R Wiedemann G. Challenges in the introduction of next-generation sequencing (NGS) for diagnostics of myeloid malignancies into clinical routine use. Blood Cancer J. (2018) 8:113. 10.1038/s41408-018-0148-6 30420667 PMC6232163

[94] Bartels S Schipper E Hasemeier B Kreipe H Lehmann U. Routine clinical mutation profiling using next generation sequencing and a customized gene panel improves diagnostic precision in myeloid neoplasms. Oncotarget. (2016) 7:30084–93. 10.18632/oncotarget.8310 27029036 PMC5058665

[95] Cross NC. Genetic and epigenetic complexity in myeloproliferative neoplasms. Hematol Am Soc Hematol Educ Progr. (2011) 2011:208–14. 10.1182/asheducation-2011.1.208 22160036

[96] Vainchenker W Dusa A Constantinescu SN. JAKs in pathology: role of Janus kinases in hematopoietic malignancies and immunodeficiencies. Semin Cell Dev Biol. (2008) 19:385–93. 10.1016/j.semcdb.2008.07.002 18682296

[97] Tefferi A Vaidya R Caramazza D Finke C Lasho T Pardanani A. Circulating interleukin (IL)-8, IL-2R, IL-12, and IL-15 levels are independently prognostic in primary myelofibrosis: a comprehensive cytokine profiling study. J Clin Oncol Offic J Am Soc Clin Oncol. (2011) 29:1356–63. 10.1200/JCO.2010.32.9490 21300928

[98] Vannucchi AM. From palliation to targeted therapy in myelofibrosis. N Engl J Med. (2010) 363:1180–2. 10.1056/NEJMe1005856 20843255

[99] Rampal R Al-Shahrour F Abdel-Wahab O Patel JP Brunel JP Mermel CH. Integrated genomic analysis illustrates the central role of JAK-STAT pathway activation in myeloproliferative neoplasm pathogenesis. Blood. (2014) 123:e123–33. 10.1182/blood-2014-02-554634 24740812 PMC4041169

[100] Barbui T Thiele J Carobbio A Passamonti F Rumi E Randi ML. Disease characteristics and clinical outcome in young adults with essential thrombocythemia versus early/prefibrotic primary myelofibrosis. Blood. (2012) 120:569–71. 10.1182/blood-2012-01-407981 22700720

[101] Haider M Gangat N Lasho T Abou Hussein AK Elala YC Hanson C. Validation of the revised International Prognostic Score of Thrombosis for Essential Thrombocythemia (IPSET-thrombosis) in 585 Mayo Clinic patients. Am J Hematol. (2016) 91:390–4. 10.1002/ajh.24293 26799697

[102] Passamonti F Giorgino T Mora B Guglielmelli P Rumi E Maffioli M. A clinical-molecular prognostic model to predict survival in patients with post polycythemia vera and post essential thrombocythemia myelofibrosis. Leukemia. (2017) 31:2726–31. 10.1038/leu.2017.169 28561069

[103] Lundberg P Karow A Nienhold R Looser R Hao-Shen H Nissen I. Clonal evolution and clinical correlates of somatic mutations in myeloproliferative neoplasms. Blood. (2014) 123:2220–8. 10.1182/blood-2013-11-537167 24478400

[104] Newberry KJ Patel K Masarova L Luthra R Manshouri T Jabbour E. Clonal evolution and outcomes in myelofibrosis after ruxolitinib discontinuation. Blood. (2017) 130:1125–31. 10.1182/blood-2017-05-783225 28674026 PMC5580275

